# p53 Protein Isoform Profiles in AML: Correlation with Distinct Differentiation Stages and Response to Epigenetic Differentiation Therapy

**DOI:** 10.3390/cells10040833

**Published:** 2021-04-07

**Authors:** Ingvild Haaland, Sigrun M. Hjelle, Håkon Reikvam, André Sulen, Anita Ryningen, Emmet McCormack, Øystein Bruserud, Bjørn Tore Gjertsen

**Affiliations:** 1 Department of Clinical Science, University of Bergen, 5021 Bergen, Norway; Ingvild.Haaland@uib.no (I.H.); Sigrun.Hjelle@uib.no (S.M.H.); Hakon.Reikvam@uib.no (H.R.); Andre.Sulen@uib.no (A.S.); Emmet.Mc.Cormack@uib.no (E.M.); oystein.bruserud@helse-bergen.no (Ø.B.); 2 Section for Hematology, Department of Medicine, Haukeland University Hospital, 5021 Bergen, Norway; 3 Chemistry and Biomedical Laboratory Sciences, Department of Safety, Faculty of Engineering and Natural Sciences, Western Norway University of Applied Sciences, 5063 Bergen, Norway; Anita.Ryningen@hvl.no; 4 Centre for Cancer Biomarkers CCBIO, Department of Clinical Science, University of Bergen, 5021 Bergen, Norway

**Keywords:** p53 protein isoforms, acute myeloid leukemia, differentiation therapy, French–American–British (FAB) classification, valproic acid, all-*trans* retinoic acid

## Abstract

p53 protein isoform expression has been found to correlate with prognosis and chemotherapy response in acute myeloid leukemia (AML). We aimed to investigate how p53 protein isoforms are modulated during epigenetic differentiation therapy in AML, and if p53 isoform expression could be a potential biomarker for predicting a response to this treatment. p53 full-length (FL), p53β and p53γ protein isoforms were analyzed by 1D and 2D gel immunoblots in AML cell lines, primary AML cells from untreated patients and AML cells from patients before and after treatment with valproic acid (VPA), all-*trans* retinoic acid (ATRA) and theophylline. Furthermore, global gene expression profiling analysis was performed on samples from the clinical protocol. Correlation analyses were performed between p53 protein isoform expression and in vitro VPA sensitivity and FAB (French–American–British) class in primary AML cells. The results show downregulation of p53β/γ and upregulation of p53FL in AML cell lines treated with VPA, and in some of the patients treated with differentiation therapy. p53FL positively correlated with in vitro VPA sensitivity and the FAB class of AML, while p53β/γ isoforms negatively correlated with the same. Our results indicate that p53 protein isoforms are modulated by and may predict sensitivity to differentiation therapy in AML.

## 1. Introduction

p53 isoforms have over the last decades emerged as promising potential biomarkers in cancer diagnosis and in therapy response prediction [1,2,3,4]. The different p53 isoforms are involved in a complex interplay, where they influence the activity of one another and regulate a variety of cellular processes [5,6,7,8]. p53 isoforms have been found to predict prognosis in cancer types like esophageal squamous cell carcinoma, prostate cancer, ovarian cancer, renal cell carcinoma, breast cancer and acute myeloid leukemia (AML) [9,10,11,12,13,14,15,16]. Although TP53 mutations are rare in AML, wild type p53 function is often inactivated through various mechanisms [17]. High levels of the p53 wild type protein have been associated with adverse karyotype and poor prognosis in therapy-related AML [18]. Induction chemotherapy in AML leads to a shift in p53 protein isoform expression, with increased p53 full length (FL) and decreased p53β/γ protein isoforms, accompanied by elevated expression of p53 target genes [19]. At diagnosis, AML patients with low levels of p53FL and high levels of p53β/γ protein isoforms seem to have a better prognosis and response to chemotherapy than patients with already high levels of p53FL and low levels of p53β/γ [16].

Differentiation therapy has been considered a promising approach for the treatment of AML for several decades, inspired by the tremendous effect of all-*trans* retinoic acid (ATRA) in the treatment of acute promyelocytic leukemia, as well as the extensive biological effects in acute myeloid leukemia cells in general [20]. However, despite the encouraging effects of several differentiation inducing agents like valproic acid (VPA) and ATRA alone and in combination in cellular models, clinical efficacy in non-acute promyelocytic leukemia remains moderate [21,22,23]. Improved knowledge about the molecular biology and therapy-induced signaling is therefore necessary to increase the efficiency of differentiation therapy.

The main aim of this work was to investigate if p53 protein isoform expression could be a potential independent biomarker for predicting a response to differentiation therapy in AML. We used 2D gel immunoblots of p53FL and p53β/γ protein isoforms and an in-house-developed pixel-by-pixel correlation method, and showed that in vitro VPA sensitivity of primary AML cells positively correlated with the expression of p53FL and negatively correlated with the expression of p53β/γ protein isoforms. 

## 2. Materials and Methods

### 2.1. Cell Lines and AML Patient Material

The human AML cell lines MOLM-13 and MV4-11 were purchased from ATCC (American Type Culture Collection, Manassas, VA, USA) and cultured according to the manufacturer’s procedure. For patient material, all studies were performed in accordance with the Helsinki declaration and approved by the Regional Ethics Committee (REC Western Norway numbers 2017/305, 2015/1410, 215.03 and 231.06). Samples were collected after informed consent, and peripheral blood mononuclear cells (PBMC) were isolated and stored frozen in liquid nitrogen as previously described [24]. Only patients with at least 80% of the leukocytes being AML cells were included in the study. The percentage of AML blasts among leukemia PBMC exceeded 95%. The clinical studies were registered in public databases (ClinicalTrials.gov no. NCT00175812 and EudraCT no. 2004-001663-22; ClinicalTrials.gov no. NCT00995332 and EudraCT no. 2007–2007–001995-36.). Clinical parameters including French–American–British (FAB) classification, cell surface markers, karyotype, resistance, survival and FLT3/NPM1 mutational status were routinely analyzed and collected. RNA extraction and p53 mutational analysis was performed as previously described [25]. 

### 2.2. Compounds

Valproic acid (Orfiril, Desitin Arzneimittel GmbH, Hamburg, Germany) (100 mg/mL in solution) was stored at −80 °C for cell culture work.

### 2.3. Western Blotting

1D and 2D gel electrophoresis and immunoblotting were performed as previously described [16,26]. The following primary antibodies were used; p53 (Bp53-12), Mdm2 (SMP-14) (Santa Cruz Biotechnology, CA, USA), Mdm2 (2A10), Mdm2 (IF2) (Calbiochem, San Diego, CA, USA), p21 (SX118) (BD Biosciences, San Jose, CA, USA), actin (AC-15) (Abcam plc, Cambridge, UK) and p53 (DO-12) (Courtesy of Dr. Jean-Christophe Bourdon, Department of Surgery and Molecular Oncology, University of Dundee, Scotland, UK), previously validated in [5,16]. The p53 (Bp53-12) and p53 (DO-12) antibodies are both mouse monoclonal antibodies. The Bp53-12 antibody binds to the N-terminus of p53, and detects p53 FL, p53β and p53γ protein isoforms. The p53 (DO-12) antibody binds to an epitope common to all p53 isoforms, and detects all p53 protein isoforms. p53 (Bp53-12) was used for 2D gel immunoblots, as this antibody gives a clear pattern for p53FL and p53β/γ isoform expression with minimal background signals. For cell lines, both p53 (Bp53-12) and p53 (DO-12) were used for 1D gel immunoblots, while only p53 (DO-12) was used for the 1D gel immunoblots for patient samples due to limited material (and in order to detect all p53 isoforms for later studies). The following secondary antibodies were used: secondary horseradish peroxidase conjugated mouse and rabbit antibodies (Jackson ImmunoResearch, West Grove, PA, USA). Regions in 1D gel immunoblots were quantified using ImageJ software version 1.53a (Wayne Rasband, National Institutes of Health, Bethesda, MD, USA).

### 2.4. Flow Cytometry

Histone acetylation, proliferation, differentiation and apoptosis markers in primary AML cells were analyzed by flow cytometry as previously described [27]. Samples were analyzed using a FACS Calibur flow cytometer (Becton Dickinson Immunocytometry Systems, San Jose, CA, USA) with an Argon laser (488 nm) and a red diode laser (635 nm). Data were analyzed using Cell Quest Lysis II software (Becton Dickinson).

### 2.5. Cell Proliferation Assay

An evaluation of proliferation in primary AML cells after drug treatment was performed using ^3^H-thymidine (Amersham International, Amersham, UK) incorporation assay as previously described [26]. Cells were cultured at 2 × 10^5^ cells/mL and treated with VPA (0 and 0.5 mM) for 48 h. ^3^H-thymidine (5 mCi in 100 mL NaCl) was added (20 µL ^3^H-thymidine to 200 µL cell suspension) 8 h before harvesting and analysis of the cells using a Packard Microplate Scintillation and Luminescence counter (PerkinElmer Life And Analytical Sciences, Inc., Waltham, MA, USA). Triplicates were analyzed for each sample.

### 2.6. Gene Expression Profiling

Global gene expression profiling of primary AML cells before and during in vivo treatment was performed as previously described [28]. All microarray experiments were performed using the Illumina iScan Reader (Illumina, San Diego, CA, USA), which is based upon fluorescence detection of biotin-labelled cRNA. Three hundred ng of total RNA from each sample was reversely transcribed, amplified and Biotin-16-UTP-labelled using the Illumina TotalPrep RNA Amplification Kit (Applied Biosystems/Ambion, Carlsbad, CA, USA). The amount and quality of the Biotin-labelled cRNA was controlled both by the NanoDrop spectrophotometer (Thermo Scientific, Wilmington, DE, USA) and Agilent 2100 Bioanalyzer (Agilent Technologies, Santa Clara, CA, USA). Biotin-labelled cRNA (750 ng) was hybridized to the HumanHT-12 V4 Expression BeadChip according to the manufacturer’s instructions. The HumanHT-12 V4 BeadChip targets 47,231 probes that are mainly derived from genes in the NCBI RefSeq database (Release 38). Data from the array scanning on Illumina iScan Reader were investigated in GenomeStudio and J-Express 2012 for quality control measures. All arrays within each experiment were quantile-normalized to be comparable before being compiled into an expression profile data matrix. Identified genes were classified by use of the PANTHER version 11 (protein annotation through evolutionary relationship) classification system, and the STRING database version 10.0 for network analyses. We have analyzed selected samples from a previous publication, where raw data are stored at Gene Expression Omnibus (accession number GSE106096). Patient numbers 1–6 (day 1/8) in our study correspond to patient numbers 31-1/31-3, 17-1/17-3, 42-1/42-3, 14-1/14-3, 23-1/23-3 and 26-1/26-3 in the stored raw data.

### 2.7. Correlation Method and Statistical Analysis

Correlation analysis was performed as previously described [16,29]. 2D gel images were aligned and normalized before Spearman rank-order correlations were performed using self-developed software. Pixel-by-pixel correlations between gel intensities and the biological variable (here, VPA response or FAB class) created a new image, showing areas in the gel image that correlated to the biological variable (the red color indicates positive correlation, while the blue color indicates negative correlation).

In addition to this main method for correlation analysis, we also performed a more traditional correlation analysis where we quantified regions of interest in 2D gel images using ImageJ software. Separate values for p53FL, p53β/γ and the ratio of p53FL:p53β/γ were obtained (signal in region of interest minus background signal in similar region), and Spearman correlations or partial correlations were performed using SPSS software (IBM SPSS Statistics version 25.0, IBM Corp., Armonk, NY, USA). Spearman correlations were performed on original values, while partial correlations were performed on log2-transformed values for p53FL, p53β/γ and the ratio of p53FL:p53β/γ. For all statistical analysis, *p <* 0.05 was considered significant. Graphs and calculations were obtained using GraphPad Prism^®^ version 6.0 (GraphPad Software, La Jolla, CA, USA) and SPSS software.

## 3. Results

### 3.1. p53 Protein Isoform Expression Correlates with In Vitro Sensitivity to Valproic Acid in Primary AML Cells

**Figure 1:**
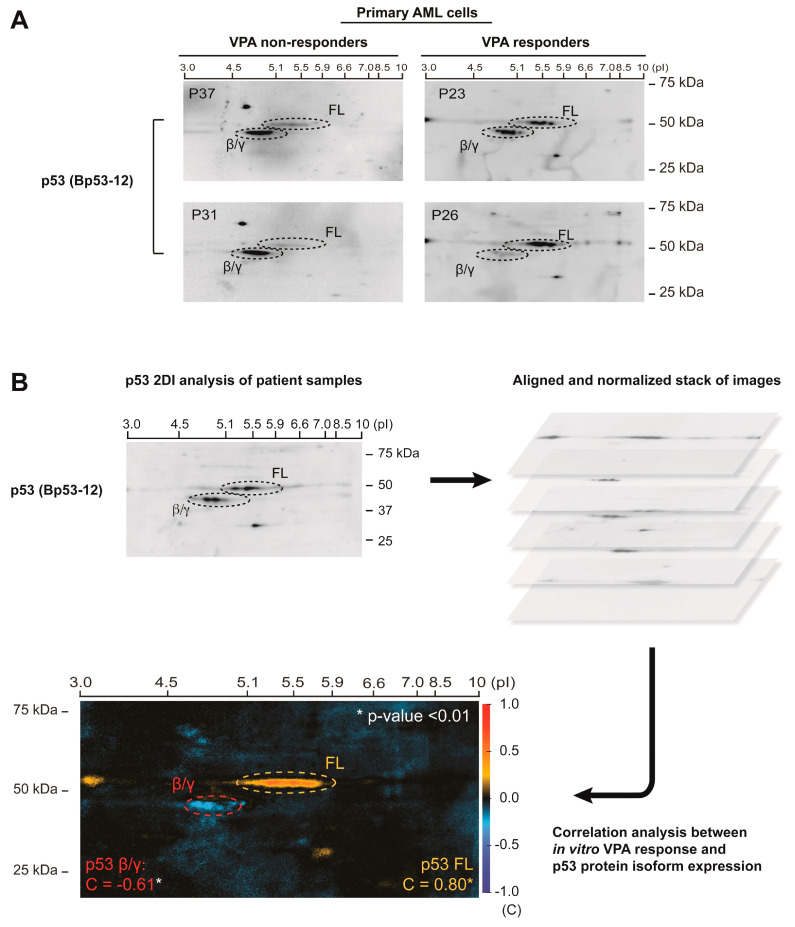
Correlation analysis between p53 protein isoform expression and in vitro sensitivity to valproic acid in primary AML cells. AML samples from 21 patients were analyzed by 2D gel electrophoresis with separation of proteins according to molecular weight (kDa) and isoelectrical point (pI), and Western blotting using an antibody against p53 (Bp53-12) for detection of p53FL, p53β and p53γ protein isoforms. In vitro VPA sensitivity was determined by treatment of cells with VPA (0 and 0.5 mM) for 48 h followed by an assessment of proliferation by ^3^H-thymidine incorporation assay. Proliferation values were calculated as % VPA response (decrease in proliferation) compared to untreated control. Examples of p53 protein isoform expression in p53 2D gel images from patient samples with low and high sensitivity to VPA are shown in (**A**). Correlation analysis between p53 protein isoform expression and VPA sensitivity was performed. Regions with significant correlation values are indicated (the red color indicates positive correlation, the blue color indicates negative correlation): β/γ region: correlation coefficient = −0.61, *t*-value = 5.50, *p*-value < 0.005, FL: correlation coefficient = 0.80, *t*-value = 5.96, *p*-value < 0.005 (**B**).

We previously developed a bioinformatic method and software to determine Spearman correlations between p53 protein isoform expression in 2D gel immunoblots and biological parameters [16,29]. In order to investigate if p53 protein isoform expression could provide independent information regarding sensitivity to differentiation therapy in AML patients, we performed a correlation analysis between p53 protein isoform expression and in vitro sensitivity to VPA in primary AML cells (PBMC with >95% AML blasts) (*n* = 21) (see Table 1 for patient characteristics). Primary AML cells were treated with 0.5 mM VPA for 48 h, followed by a determination of proliferation (^3^H-thymidine incorporation), while an analysis of p53 isoforms was performed in untreated samples. The samples were analyzed by p53 2D gel immunoblotting (detecting p53 FL, p53β and p53γ isoforms), images were aligned and normalized and correlation analysis was performed as previously described [16]. Examples of p53 protein isoform expression in patient samples with high and low sensitivity to VPA in vitro are shown in Figure 1A. The correlation analysis demonstrated a significant positive correlation between VPA sensitivity and the expression of p53 FL (correlation coefficient = 0.80, *t*-value = 5.96, *p*-value < 0.005), and a significant inverse correlation between VPA sensitivity and the expression of p53β/γ isoforms (correlation coefficient = −0.61, *t*-value = 5.50, *p*-value < 0.005) (Figure 1B) (See Supplementary Table S1 and Supplementary Figure S1-1 values for VPA sensitivity and 2D gel images). This indicates that the more sensitive patient samples would express high levels of p53 FL and low levels of p53β/γ isoforms.

As a supplement to this self-developed correlation analysis, we also quantified regions of interest in 2D gel images using ImageJ software. Spearman correlations in SPSS demonstrated significant positive correlations between the VPA response and p53FL (*r* = 0.465, *p* = 0.039) and between the VPA response and the ratio of p53FL:p53β/γ (*r* = 0.673, *p* = 0.001). No significant correlation was found between the VPA response and p53β/γ (*r* = −0.071, *p* = 0.767). Pearson correlations using log2-transformed values for p53FL, p53β/γ and the ratio of p53FL:p53β/γ demonstrated significant correlation only between the VPA response and the ratio (*r* = 0.610, *p* = 0.004). Furthermore, partial correlations between the VPA response and the ratio demonstrated significant positive correlations when controlling for p53FL (*r* = 0.513, *p* = 0.025), and when controlling for p53β/γ (*r* = 0.574, *p* = 0.010) (See Supplementary Figure S1-2).

### 3.2. p53 Protein Isoform Expression Correlates with the Differentiation Stage of AML Blasts

**Figure 2:**
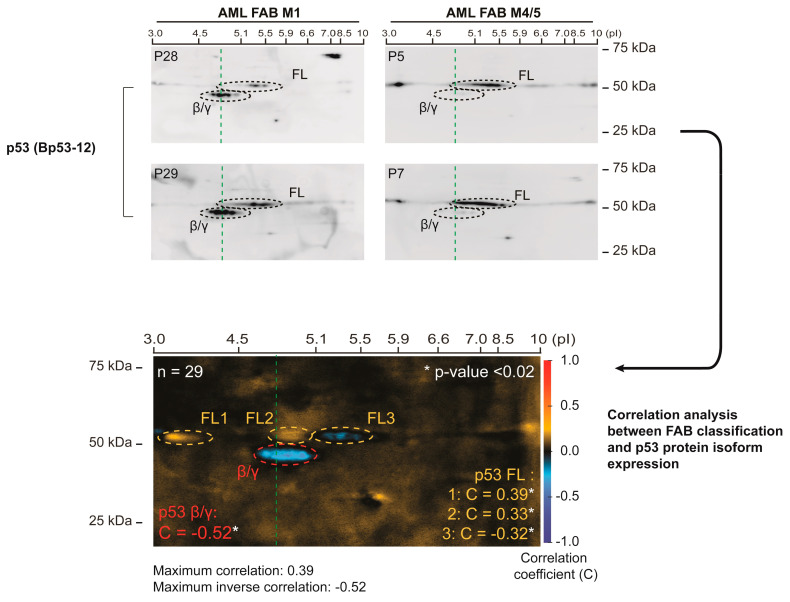
Correlation analysis between p53 protein isoform expression and morphological differentiation stage (French–American–British classification) of AML blasts. 29 primary AML samples with defined FAB classification were analyzed by 2D gel electrophoresis and Western blotting using an antibody against p53 (Bp53-12) for the detection of p53 full length (FL), p53β and p53γ protein isoforms. Examples of p53 protein isoform expression in p53 2D gel images from patient samples of low and high FAB class (M1 versus M4/5) are shown. A correlation analysis between p53 protein isoform expression and FAB classification (patient samples were assigned values from 0-6 based on FAB class) of the AML blasts was performed. Regions with significant correlation values are indicated (the red color indicates positive correlation, the blue color indicates negative correlation): β/γ region: correlation coefficient = −0.52, *t*-value = 4.51, *p*-value < 0.005), FL1: correlation coefficient = 0.39, *t*-value = 3.14, *p*-value < 0.005, FL2: correlation coefficient = 0.33, *t*-value = 2.59, *p*-value < 0.02), FL3: correlation coefficient = −0.32, *t*-value = 2.50, *p*-value < 0.02.

We performed a correlation analysis between p53 protein isoform expression and French–American–British (FAB) classification (morphology and differentiation stage) in 29 AML patient samples previously not analyzed (see Table 2 for patient characteristics). Examples of p53 protein isoform expression in patient samples from patients with FAB M1 (low level of differentiation) versus patients with FAB M4/5 (high level of differentiation) are shown in Figure 2. The correlation analysis was carried out as described above, and demonstrated a significant inverse correlation between FAB classification and the p53β/γ region (correlation coefficient = −0.52, *t*-value = 4.51, *p*-value < 0.005), indicating that AML patient samples of a less differentiated phenotype would express higher levels of p53β/γ isoforms than the more differentiated phenotype (higher FAB classification). Furthermore, significant positive correlations were observed for the more acidic (lower pI) regions of p53 FL (FL1: correlation coefficient = 0.39, *t*-value = 3.14, *p*-value < 0.005, FL2: correlation coefficient = 0.33, *t*-value = 2.59, *p*-value < 0.02), and a significant negative correlation was observed for the less acidic region of p53 FL (FL3: correlation coefficient = −0.32, *t*-value = 2.50, *p*-value < 0.02), indicating that patient samples with higher differentiation stage would express higher levels of more acidified (putatively phosphorylated and activated) forms of p53 FL (more detailed information is in Supplementary Table S1 and Supplementary Figure S2).

Additional correlation analyses were performed with the quantification of regions of interest in 2D gel images using ImageJ software and Spearman correlation analysis in SPSS. The results demonstrated a significant negative correlation between the FAB class and p53β/γ (*r* = −0.4294, *p* = 0.023), and no significant correlations between the FAB class and p53FL (*r* = 0.057, *p* = 0.775) or between the FAB class and the ratio of p53FL:p53β/γ (*r* = 0.2719, *p* = 0.162).

### 3.3. p53 Protein Isoforms Are Modulated by Valproic Acid in AML Cell Lines

**Figure 3:**
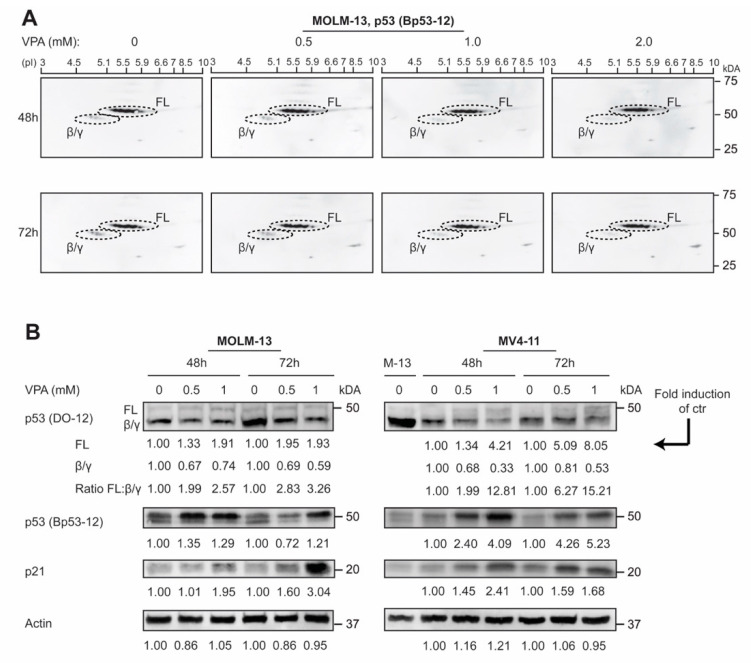
Valproic acid-induced modulation of p53 protein isoforms in AML cell lines. (**A**) The AML cell line MOLM-13 (AML FAB M5a) was treated with VPA (0, 0.5, 1.0 and 2.0 mM) for 48 and 72 h, and analyzed by 2D gel electrophoresis and Western blotting for the detection of p53 full-length (FL), p53β and p53γ protein isoforms (Bp53-12). (**B**) AML cell lines MOLM-13 and MV4-11 (AML FAB M5) were treated with VPA (0, 0.5, and 1.0 mM) for 48 and 72 h and analyzed by 1D gel electrophoresis and Western blotting using antibodies against p53 (DO-12), p53 (Bp53-12) and p21. Actin was used as a loading control. Regions in Western blots (p53 DO-12) were quantified, and ratios of p53 FL: p53β/γ were calculated and given as fold induction of control.

Considering the observed correlation between p53 protein isoform expression and the differentiation stage of AML blasts described above, we investigated the modulation of p53 isoform expression in differentiation therapy of AML. The AML cell lines MOLM-13 (AML FAB M5a) and MV4-11 (AML FAB M5) were treated with VPA (0, 0.5, 1 and 2 mM) for 48 and 72 h, followed by 2D gel immunoblot analysis with an antibody against p53 (Bp53-12; detects p53 FL, p53β and p53γ) (Figure 3A), or 1D gel immunoblot analysis using antibodies against p53 (DO-12; detects all p53 isoforms), p53 (Bp53-12), p21 and actin (Figure 3B). p53 2D gel immunoblots showed the same pattern as for the correlation analysis, with downregulation of p53β/γ isoforms in more differentiated cells (those treated with VPA), while 1D gel immunoblots also demonstrated upregulation of p53 FL concomitant with the downregulation of p53β/γ isoforms after VPA treatment in both cell lines, similar to the expression pattern of patient samples with a high level of differentiation from Figure 2 (for details, see Supplementary Figure S3-1 and 3-2). 

### 3.4. In Vivo Modulation of p53 Protein Isoforms during Differentiation Therapy of AML

**Figure 4:**
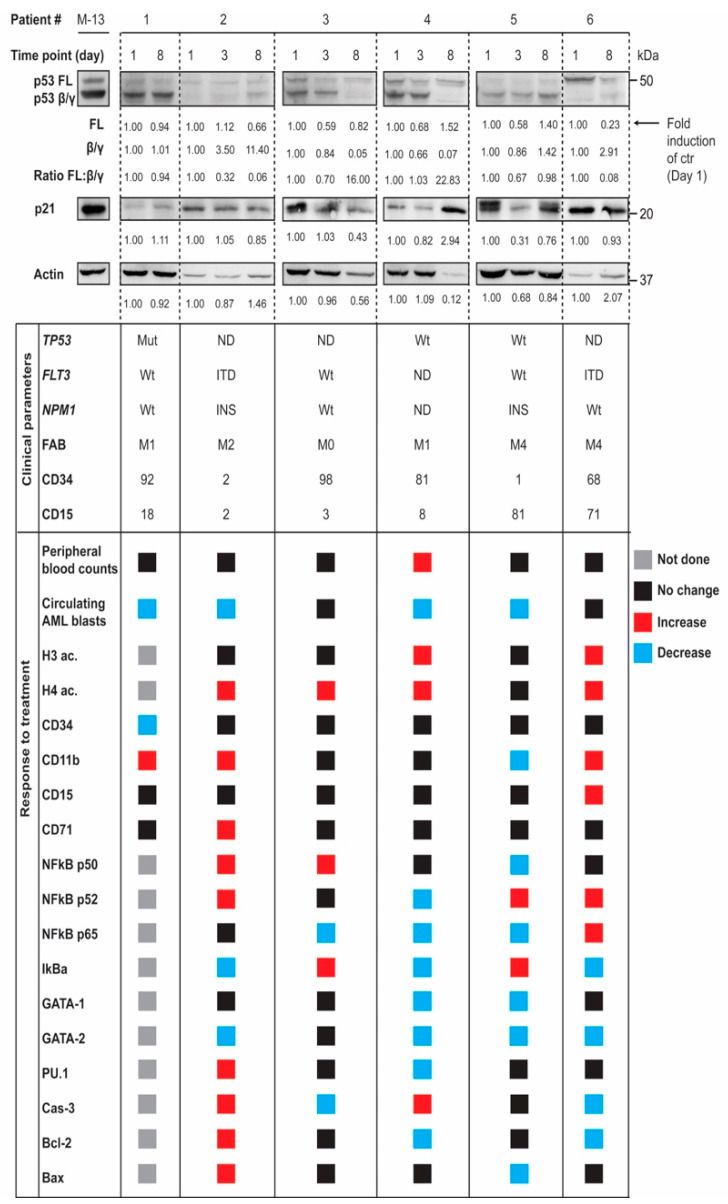
In vivo modulation of p53 protein isoforms during differentiation therapy in AML. Samples were collected from AML patients undergoing combination therapy of valproic acid (VPA), all-*trans* retinoic acid (ATRA) and theophylline before treatment (day 1), after treatment with ATRA (day 3) and after treatment with the triple combination (day 8). Samples were analyzed by 1D gel electrophoresis and Western blotting with antibodies against p53 (DO-12), p21 and actin. Regions in Western blots were quantified and ratios of p53 FL: p53β/γ were calculated and given as fold induction of control (day 1). Clinical parameters including *TP53, FLT3* and *NPM1* status, FAB classification, CD34, CD15 and response to treatment, including peripheral blood counts and circulating AML blasts are shown below. Histone acetylation, proliferation, differentiation and apoptosis markers are analyzed by flow cytometry. Results are based on median fluorescence intensity (MFI), except from CD markers and cell cycle markers, where results are based on % positive cells. Response to treatment is marked grey for not done, black for no change, red for increase and blue for decrease. Abbreviations: ac, acetylation; Bax, Bcl-2-like protein 4; Bcl-2, B-cell lymphoma; CD, cluster of differentiation; FAB, French–American–British; FL, full-length; FLT3, FMS-like tyrosine kinase 3; GATA, globin transcription factor; H3, Histone H3; H4, Histone H4; IkBa, nuclear factor of kappa light polypeptide gene enhancer in B-cells inhibitor, alpha; ITD, Internal tandem duplication; MDS, myelodysplastic syndrome; ND, not done; NFkB, nuclear factor kappa-light-chain-enhancer of activated B cells; Wt, Wild type.

We investigated p53 isoform expression in patient material from a clinical protocol where patients were treated with oral ATRA 22.5 mg/m^2^ twice daily for the first 14 days, and VPA together with theophylline from day 3 until disease progression, as previously described [27]. Samples were collected before treatment (day 1), after 2 days of treatment with ATRA alone (day 3) and after 5 additional days of treatment with the triple combination (day 8). 1D gel immunoblots of p53 isoform expression (detected by p53 DO-12) from the 6 different patients at different time points during treatment (day 1, 3 and 8) demonstrated a modulation of p53 isoform expression also in vivo during differentiation therapy (Figure 4).

Two of the patients responded with downregulation of p53β/γ isoforms on day 8, similar to AML cell lines treated with VPA in vitro, one of them also accompanied by a significant increase in p21. As patient material may differ in levels of actin and other commonly used loading controls, the ratio between p53 FL and p53β/γ isoform expression was calculated, demonstrating an increased p53 FL: p53β/γ ratio in some of the samples during treatment. Clinical parameters of the patient material and response to treatment are described below, and were determined previously [27] (see Supplementary Table S2 and Supplementary Figure S4 for more detailed characteristics).

A gene expression profiling analysis was performed for the same patients before and after therapy (Supplementary Figures S5 and S6). Distinct gene expression profiles were identified after treatment. Functional network analysis revealed two protein networks of special interest; (i) the network related to proliferating cell nuclear antigen (PCNA), and (ii) protein interactions related to phosphatase and tensin homolog (PTEN). Both protein networks are connected to the p53 pathway at various levels of regulation [30,31,32,33,34].

## 4. Discussion

The observed correlation between p53 protein isoform expression and in vitro VPA sensitivity in primary AML cells indicates that patients with high levels of p53FL and low levels of p53β/γ would be more sensitive to treatment with VPA. Several reports suggest that high expression of p53FL, low expression of p53β/γ isoforms and a high ratio of p53FL relative to p53β/γ isoforms are associated with an adverse prognosis in leukemia patients [16,18]. The fact that patients with this adverse p53 expression pattern were the most sensitive to VPA treatment suggests that this therapy may be beneficial in particular for patients with poor prognosis. This is promising for further development of differentiation therapy, a therapy approach suggested to provide clinical benefit in high-risk AML patients [21,22,23,27]. However, the results need to be validated in larger studies, ideally with correlative analysis of p53 isoform expression with response to in vivo differentiation therapy.

p53 protein isoform expression also correlated with FAB classification (differentiation stage) of AML, indicating that patient AML blasts with a low level of differentiation would express high levels of p53β/γ isoforms compared to patients with a high level of differentiation. This is consistent with findings of Hofstetter and colleagues, who reported an association between expression of p53β with poorly differentiated ovarian cancers [11]. In this study, p53β expression also correlated with poor recurrence-free and overall survival. Conversely, AML patients with elevated expression levels of p53β/γ protein isoforms demonstrated improved responses to chemotherapy and increased overall survival [16]. Furthermore, breast cancer patients expressing p53γ together with mutated p53 had a prognosis as good as those expressing wild type p53 [15], and high levels of p53β were associated with disease-free survival in breast cancer [14]. These studies and our results suggest that the p53 isoforms are differentially expressed in cancer cells of various differentiation stages and may predict prognosis and therapy response; however, this may differ according to cancer type and type of therapy.

The advantage of the self-developed pixel-by-pixel correlation method is that it is sensitive and gives a detailed correlation for specific parts of the p53 protein isoform profile. This method showed a stronger correlation between the VPA response and p53FL compared to the VPA response and p53β/γ (correlation coefficients 0.80 vs. −0.61). The alternative method used for correlation analysis (quantification with ImageJ and Spearman or partial correlations) was not as sensitive. However, we could also calculate correlations between the VPA response and the ratio of p53FL: p53β/γ using this method. The ratio was a stronger predictor of the VPA response compared to p53FL (correlation coefficients 0.610 vs. 0.384), and remained significant also after controlling for p53FL or p53β/γ. However, p53FL did influence the relationship between the VPA response and the ratio of p53FL: p53β/γ to some degree.

In AML cell lines expressing wild type p53, there was a marked downregulation of p53β/γ isoforms after VPA treatment, as well as an upregulation of p53FL. Interestingly, a modulation of p53 protein isoforms was also detected in vivo during differentiation therapy. A noticeable downregulation of p53β/γ isoforms was observed in some of the patients, while the increase in p53FL was (as expected) not as evident as for in vivo chemotherapy [16]. p53FL and p53β/γ isoforms are shown to participate in the regulation of cancer development and in cellular processes like differentiation and apoptosis [35,36], and p53β/γ isoforms have been found to enhance both p53FL functions and p53-independent chemosensitivity [5,37,38]. In AML cells with already activated p53 (cells treated with chemotherapy or cells with a high level of differentiation), it is possible that differentiation therapy could be sufficient to induce differentiation or apoptosis in a p53-dependent manner. Combination therapy focusing on modulating p53 isoform expression to a more favorable profile prior to differentiation therapy could therefore be feasible [39].

## 5. Conclusions

Altogether, our results demonstrate that p53 isoform expression is modulated by differentiation therapy in AML, both in vitro and in vivo, and that p53 isoform expression could affect sensitivity to differentiation-inducing agents. We anticipate that p53 protein isoform expression analysis may add independent information on predicting therapy response. Future studies should include a correlation analysis of p53 isoform expression with response to in vivo differentiation therapy, as well as explore the potential of p53 isoform-targeted therapy in aggressive blood cancers like AML.

## Figures and Tables

**Table 1:** Acute myeloid leukemia (AML) patient characteristics (in vitro valproic acid (VPA) response correlation).

Age and Gender		Fab Classification	
Median age (years)	63	M0/1	6
Range age (years)	29/82	M2	4
Female	11	M3	1
Male	12	M4/5	10
Total	21		
**Previous Malignancies**		**Cytogenetics**	
MDS	2	Adverse	3
CML, recidiv	1	Favorable	2
PVR	1	Intermediate	11
MDS, AML relapse	1	Unknown	5
Cancer ovarii	1		
**Mutations**		**CD-markers**	
**NPM1 mutations**		CD13 neg	1
Mut	4	pos	16
WT	6	CD14 neg	15
n.d.	11	pos	2
**FLT3 mutations**		CD15 neg	12
ITD	4	pos	5
WT	14	CD33 neg	0
n.d.	3	pos	17
**TP53 mutations**		CD34 neg	6
Mut (del)	2	pos	11
WT	17	n.d.	3
n.d.	2		
**Disease res.**		**Survival (months)**	
Yes	7	Median survival	4
No	4	Survival range	0–36
n.d.	10		
**Valproic acid response (%)**			
Median response	8.5		
Range response	−55.6–48.6		

**Abbreviations:** CD, Cluster of differentiation; Del, Deletion; Disease res, Resistant disease; FAB, French-American-British classification of acute myeloid leukemia M0-M6; FLT3, FMS-like tyrosine kinase 3; ITD, Internal tandem duplication; MDS, Myelodysplastic syndrome; Nd, Not determined; NPM1, Nucleophosmin 1; PVR, Polycythemia vera; WT, Wild-type. In vitro valproic acid response was determined by ^3^H-thymidine proliferation assay, and given as % change compared to untreated control.

**Table 2:** AML patient characteristics (French–American–British (FAB) correlation).

Age and Gender		Fab Classification	
Median age (years)	64	M0/1	11
Range age (years)	34-82	M2	5
Female	13	M3	0
Male	16	M4/5	12
Total	29	M6	1
**Previous Malignancies**		**Cytogenetics**	
MDS	6	Adverse	5
CML, recidiv	1	Favorable	1
AML, recidiv	1	Intermediate	11
PVR	1	Unknown	12
MDS, AML relapse	1		
**Mutations**		**CD-markers**	
**NPM1 mutations**		CD13 neg	3
Mut	3	pos	22
WT	14	CD14 neg	18
n.d.	12	pos	6
**FLT3 mutations**		CD15 neg	10
ITD	10	pos	11
WT	15	CD33 neg	6
n.d.	4	pos	19
**TP53 mutations**		CD34 neg	8
Mut (del)	2	pos	13
WT	22	n.d.	4
n.d.	5		
**Disease res.**		**Survival (months)**	
Yes	5	Median survival	5
No	7	Survival range	0–72
n.d.	17		

**Abbreviations:** CD, Cluster of differentiation; Del, Deletion; Disease res, Resistant disease; FAB, French-American-British classification of acute myeloid leukemia M0-M6; FLT3, FMS-like tyrosine kinase 3; ITD, Internal tandem duplication; MDS, Myelodysplastic syndrome; Nd, Not determined; NPM1, Nucleophosmin 1; PVR, Polycythemia vera; WT, Wild-type.

## Data Availability

The data presented in this study are available within the article and Supplementary Materials.

## Supplementary Materials

The following are available online at https://www.mdpi.com/article/10.3390/cells10040833/s1, Figure S1-1: Details for data used in Figure 1, Figure S1-2: Partial correlation analysis between VPA response and the ratio of p53FL:p53β/γ in AML blasts, Figure S2: Details for data used in Figure 2, Figure S3-1: Details for data used in Figure 3A, Figure S3-2: Details for data used in Figure 3B, Figure S4: Details for data used in Figure 4, Figure S5: Gene expression profiling in AML after treatment with ATRA, valproic acid and theophylline, Figure S6: Network analysis based on gene expression profiling data, Table S1: Data used for correlation analysis, Table S2: Characteristics of AML patients included in the clinical protocol.
