# Supplementary material for: p53 Protein Isoform Profiles in AML: Correlation with Distinct Differentiation Stages and Response to Epigenetic Differentiation Therapy
Source: Cells. 2021 Apr 7;10(4):833. doi: 10.3390/cells10040833 (PMC8068061; doi:10.3390/cells10040833)
Supplement: Supplementary file 1 [file cells-10-00833-s001.zip › Supplementary data for paper/Supplementary Figure 1-2 with text.pdf]

A VPA response vs ratio

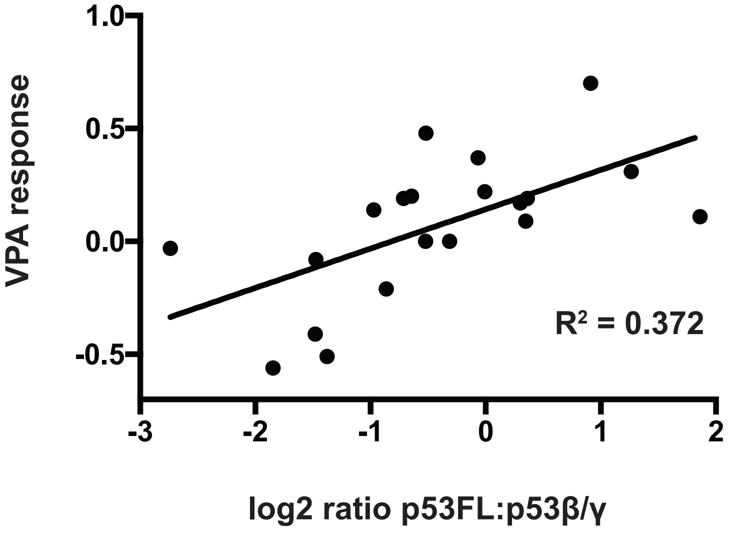

B VPA response vs ratio when controlling for p53FL

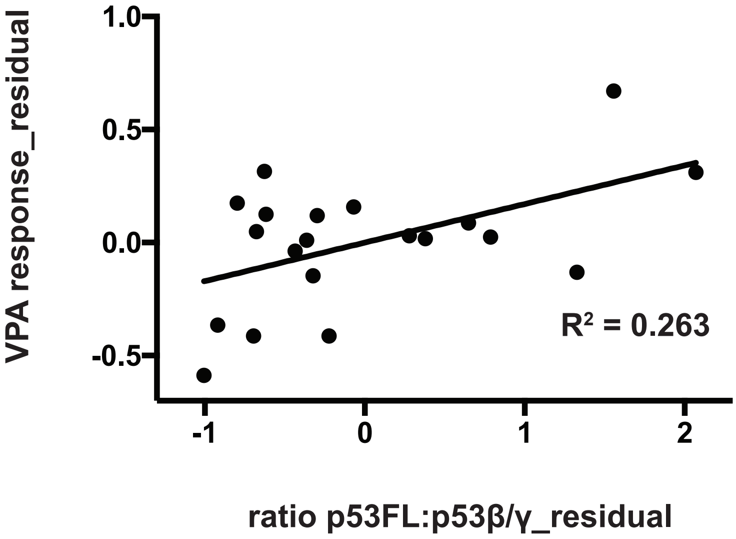

C VPA response vs ratio when controlling for p53β/γ

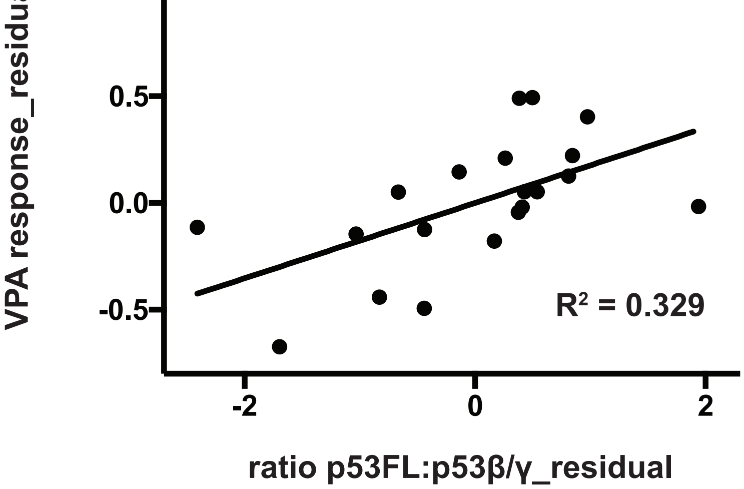

Supplementary Figure 1-2. Partial correlation analysis between VPA response and the ratio of p53FL:p53β/γ in AML blasts. AML samples from 21 patients were analyzed by 2D gel electrophoresis and Western blotting using an antibody against p53 (Bp53-12) for detection of p53FL, p53β and p53γ protein isoforms. In vitro VPA sensitivity was determined by treatment of cells with VPA (0 and 0.5 mM) for 48 hours followed by assessment of proliferation by ^{3}H-thymidine incorporation assay. Proliferation values were calculated as % VPA response (decrease in proliferation) compared to untreated control (percent values were converted to decimals). Regions in 2D gel images were quantified using ImageJ software, and values obtained for p53FL, p53β/γ and the ratio of p53FL:p53β/γ were log2-transformed (one blot was removed from the dataset due to negative quantified value for p53FL). Figure A shows the Pearson correlation plot between VPA response and the ratio of p53FL:p53β/γ. Figure B shows the partial correlation plot between VPA response and the ratio when controlling for p53FL, and Figure C shows the partial correlation plot between VPA response and the ratio when controlling for p53 β/γ.
