# Supplementary material for: p53 Protein Isoform Profiles in AML: Correlation with Distinct Differentiation Stages and Response to Epigenetic Differentiation Therapy
Source: Cells. 2021 Apr 7;10(4):833. doi: 10.3390/cells10040833 (PMC8068061; doi:10.3390/cells10040833)
Supplement: Supplementary file 1 [file cells-10-00833-s001.zip › Supplementary data for paper/Supplementary Figure 5 with text.pdf]

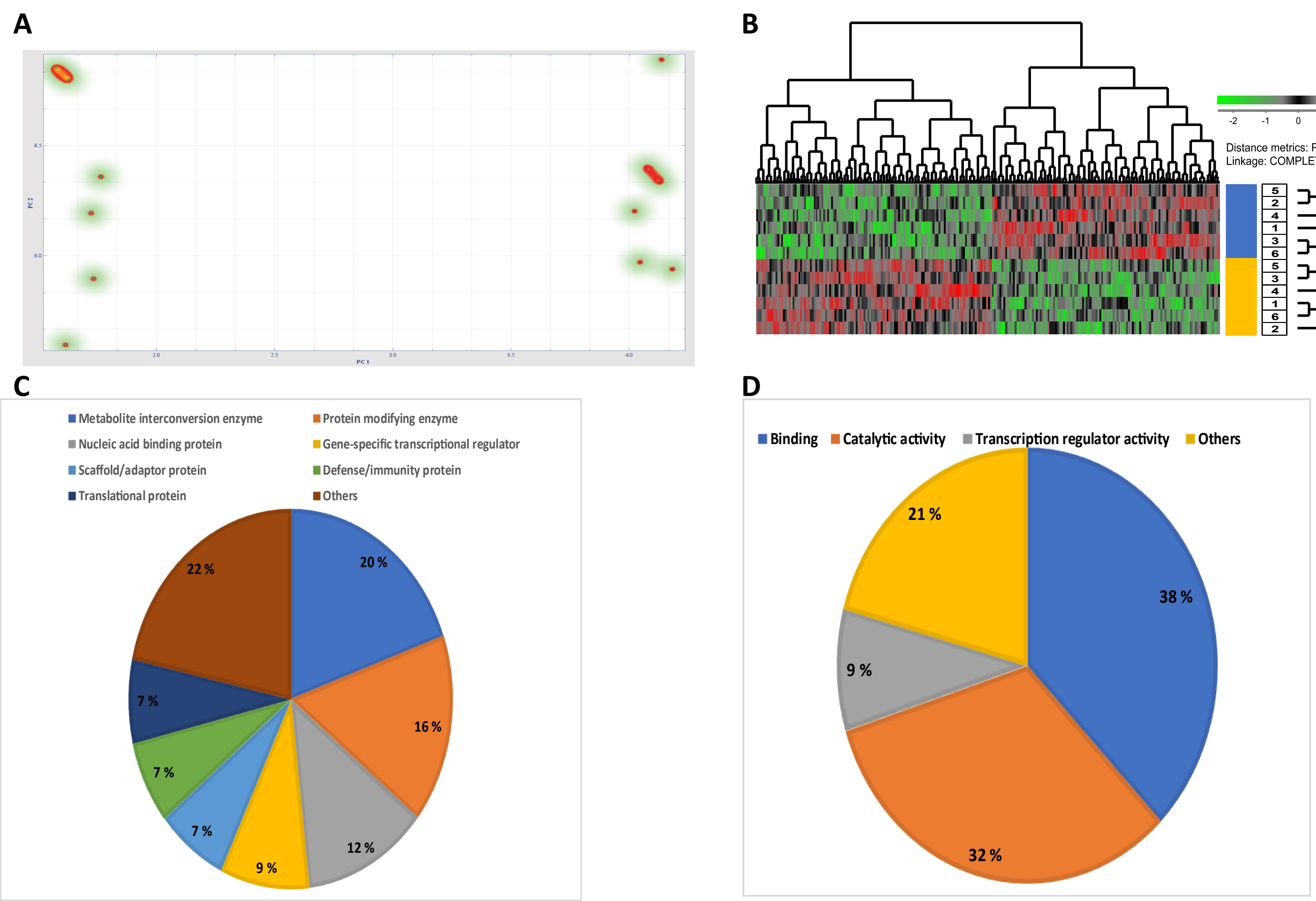
A
B
-2
-1
0
1
2
Distance metrics: Pearson Correlation
Linkage: COMPLETE
5
2
4
1
3
6
5
3
4
1
6
2
C
Metabolite interconversion enzyme
Protein modifying enzyme
Nucleic acid binding protein
Gene-specific transcriptional regulator
Scaffold/adaptor protein
Defense/immunity protein
Translational protein
Others
20 %
16 %
12 %
9 %
7 %
7 %
7 %
22 %
D
Binding
Catalytic activity
Transcription regulator activity
Others
38 %
32 %
9 %
21 %

S5

Supplementary Figure 5. Gene expression profiling in AML after treatment with ATRA, valproic acid and theophylline. Samples used for gene expression profiling (GEP) analyses were collected before treatment (day 1), and after 7 days of treatment with the triple combination (day 8). GEP data for six patients enrolled in the clinical part of the study were available, and 127 genes were found differently expressed (F score of +/- 1.6). **(A)** Principal component analyses based on the differently expressed genes were highly able to discriminate the patients before and after treatment. **(B)** Unsupervised hierarchical clustering analysis (Pearson correlation as distance measure and complete linkage) based on the 127 differently expressed genes. Red bricks indicate upregulated genes and green downregulated genes. Blue column bar indicates patients before treatment and yellow after treatment. **(C)** The PANTHER classification system based on the ontology protein class are demonstrated for the proteins based on the encoding genes from the 127 genes identified differently expressed. **(D)** The PANTHER classification system based on the ontology molecular function are demonstrated for the proteins based on the encoding genes from the 127 genes identified differently expressed.
