# Supplementary material for: p53 Protein Isoform Profiles in AML: Correlation with Distinct Differentiation Stages and Response to Epigenetic Differentiation Therapy
Source: Cells. 2021 Apr 7;10(4):833. doi: 10.3390/cells10040833 (PMC8068061; doi:10.3390/cells10040833)
Supplement: Supplementary file 1 [file cells-10-00833-s001.zip › Supplementary data for paper/Supplementary Figure 6 with text.pdf]

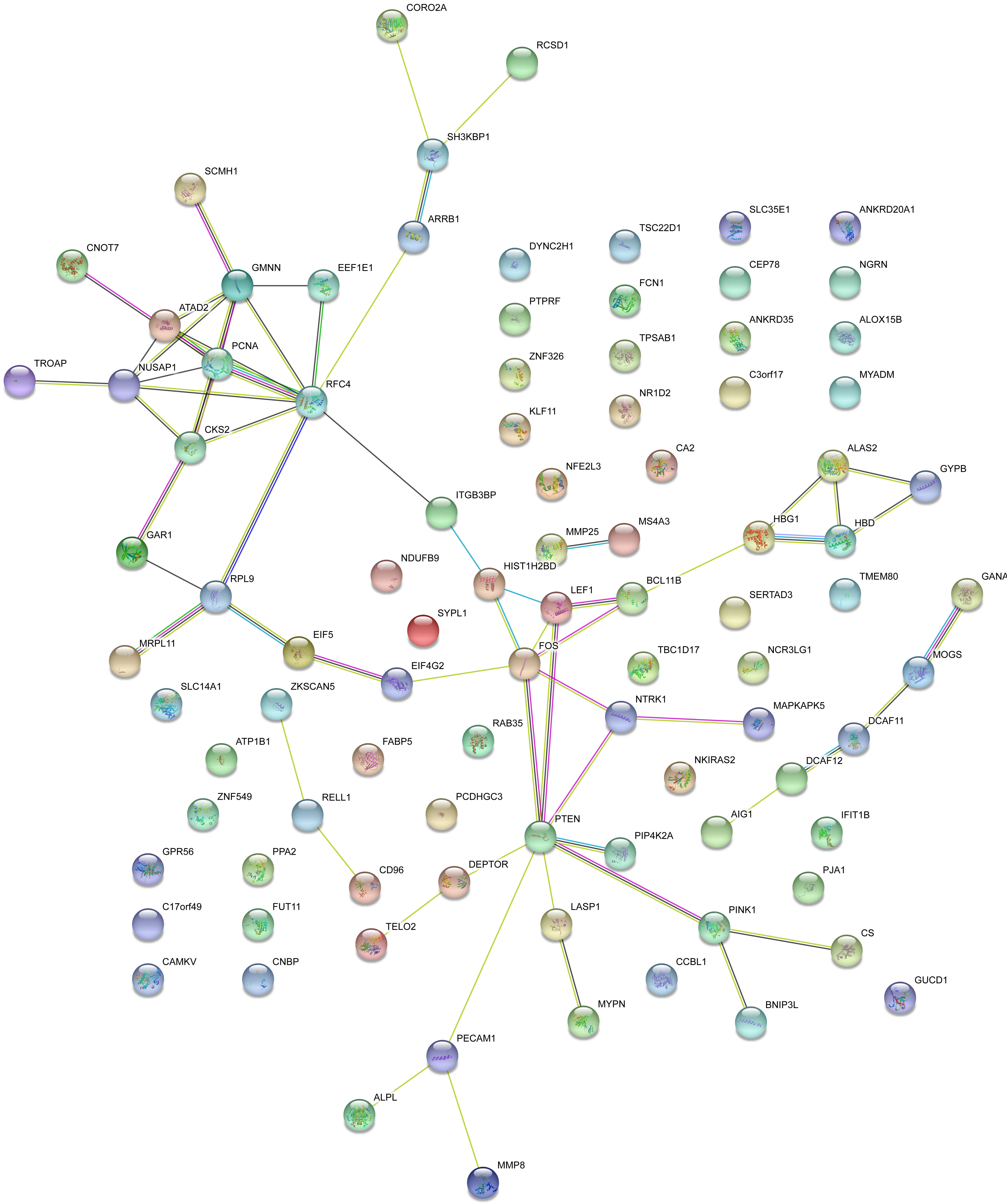

CORO2A
RCSD1
SH3KBP1
SCMH1
ARRB1
SLC35E1
ANKRD20A1
TSC22D1
CNOT7
DYNC2H1
GMNN
EEF1E1
CEP78
NGRN
FCN1
PTPRF
ATAD2
ANKRD35
ALOX15B
TPSAB1
PCNA
ZNF326
TROAP
NUSAP1
C3orf17
MYADM
RFC4
NR1D2
KLF11
CKS2
CA2
ALAS2
NFE2L3
GYPB
ITGB3BP
MS4A3
HBG1
MMP25
HBD
GAR1
NDUFB9
HIST1H2BD
TMEM80
GANA
BCL11B
RPL9
LEF1
SERTAD3
SYPL1
MRPL11
EIF5
FOS
TBC1D17
NCR3LG1
MOGS
EIF4G2
SLC14A1
ZKSCAN5
NTRK1
MAPKAPK5
DCAF11
RAB35
ATP1B1
FABP5
NKIRAS2
DCAF12
ZNF549
RELL1
PCDHGC3
AIG1
IFIT1B
PTEN
PIP4K2A
GPR56
PPA2
DEPTOR
CD96
PJA1
C17orf49
FUT11
LASP1
PINK1
TELO2
CS
CAMKV
CNBP
CCBL1
GUCD1
MYPN
BNIP3L
PECAM1
ALPL
MMP8

Supplementary Figure 6. Network analysis based on gene expression profiling data. We performed a network analysis using the STRING database for analyzing the functional interactions between the proteins encoded by the genes found differently expressed after treatment. The figure demonstrates potential interactions between the proteins encoded by the genes found differently expressed. Based on this analysis we identified two central protein network of special interest; (i) the network related to proliferating cell nuclear antigen (PCNA) and (ii) protein interactions related to phosphatase and tensin homolog (PTEN). Both these protein networks are believed to be central in several process related to malignant disease development and maintenance.
