# Supplementary material for: p53 Protein Isoform Profiles in AML: Correlation with Distinct Differentiation Stages and Response to Epigenetic Differentiation Therapy
Source: Cells. 2021 Apr 7;10(4):833. doi: 10.3390/cells10040833 (PMC8068061; doi:10.3390/cells10040833)
Supplement: Supplementary file 1 [file cells-10-00833-s001.zip › Supplementary data for paper/Supplementary table 1.pdf]

Supplementary Table 1: Data used for correlation analysis

Patient nr	FAB correlation value	VPA respose correlation value
1	4	
2	2	
3	1	
4	4	
5	4	
6	0,5	
7	5	
8	4	
9	0,5	
10	1	
11	6	
12	1	
13	4	
14	4	
15	2	
16	5	
17	5	
18	2	
19	2	0,14
20	1	0,37
21	0	0,20
22	4	0,17
23	1	0,22
24	2	0,31
25	4	-0,08
26	4	0,11
27	1	0,00
28	1	0,19
29	1	-0,51
30		0,09
31		-0,56
32		-0,03
33		-0,41
34		0,00
35		0,48
36		-0,13
37		-0,21
38		0,19
39		0,7
