# Supplementary material for: p53 Protein Isoform Profiles in AML: Correlation with Distinct Differentiation Stages and Response to Epigenetic Differentiation Therapy
Source: Cells. 2021 Apr 7;10(4):833. doi: 10.3390/cells10040833 (PMC8068061; doi:10.3390/cells10040833)
Supplement: Supplementary file 1 [file cells-10-00833-s001.zip › Supplementary data for paper/Supplementary Table 2.pdf]

Supplementary Table 2: Characteristics of AML patients included in the clinical protocol

		Protocol, Patient #					
**CD markers**		**1**	**2**	**3**	**4**	**5**	**6**
CD13		58	4	99	81	20	40
CD14		1	1	1	1	4	8
CD15		18	2	3	8	81	71
CD33		35	99	11	45	98	89
CD34		92	2	98	81	1	68
**Response to therapy**	**Day**	**1**	**2**	**3**	**4**	**5**	**6**
LPK (x10^9/l)	1	34	217	13	142	73	66
	8	55	173	15	113	48	137
TPK (x10^9/l)	1	14	27	111	16	24	32
	8	16	7	90	15	11	16
Later response		No later response	No later response	No later response	TPK incr., LPK red. to 15%	No later response	No later response
Histon H3	1	ND	30.2	17.5	19.2	8.9	21.4
	3	ND	29.1	25.6	18.1	7.2	22.7
	8	ND	30.7	18.6	27.1	7.4	29.7
Histon H4	1	ND	20.5	16.2	19.7	10.1	16.2
	3	ND	25.2	23.4	15.2	10.2	23.4
	8	ND	24.1	23.6	22.5	9.6	23.6
NFkB, p50	1	ND	16.7	14.7	20.1	44.4	27.0
	3	ND	18.8	16.1	17.0	34.5	18.8
	8	ND	19.2	18.2	19.7	37.3	25.8
NFkB, p52	1	ND	23.5	15.8	23.1	21.0	26.9
	3	ND	20.7	20.8	19.6	23.6	26.4
	8	ND	25.9	17.2	20.3	33.7	29.9
NFkB, p65	1	ND	28.7	51.4	55.2	54.8	53.8
	3	ND	26.3	43.8	35.8	29.3	54.6
	8	ND	26.4	42.8	36.9	47.3	71.0
IKBa	1	ND	49.0	14.6	40.4	28.4	35.6
	3	ND	33.7	22.3	11.7	37.0	29.5
	8	ND	43.0	30.0	8.0	39.3	30.7
Cell cycle G0/G1	1	ND	95.7	19.8	91.7	95.3	90.4
	3	ND	93.9	29.5	94.8	85.6	91.0
	8	ND	94.7	23.1	97.1	89.4	83.2
Cell cycle S	1	ND	1.7	0.6	0.9	1.7	2.6
	3	ND	3.3	0.3	0.9	2.6	3.1
	8	ND	3.8	0.7	0.6	2.6	7.5
Cell cycle G2/M	1	ND	1.2	77.2	6.5	1.8	5.0
	3	ND	2.0	69.3	3.3	5.7	3.6
	8	ND	0.9	74.7	1.6	4.4	4.3
CD34	1	95.6	7.4	67.2	91.3	3.3	69.6
	3	86.1	3.2	64.0	89.7	3.1	70.6
	8	85.2	1.3	68.6	86.0	3.7	72.6
CD11b	1	8.1	28.7	9.1	4.7	44.0	31.6
	3	10.7	25.3	9.5	8.5	41.6	39.6
	8	24.2	52.8	10.3	12.4	25.0	53.1
CD15	1	2.3	2.0	0.3	3.0	37.2	10.4
	3	6.9	0.6	0.3	5.8	39.2	16.5
	8	11.4	0.3	0.2	4.8	30.5	21.3

		Protocol, Patient #					
**Response to therapy**	**Day**	**1**	**2**	**3**	**4**	**5**	**6**
CD71	1	23.3	41.2	40.8	11.3	1.6	8.2
	3	12.9	35.8	42.8	8.2	0.8	3.9
	8	17.1	66.1	41.7	10.8	1.2	3.4
GATA-1	1	ND	33.1	25.7	20.8	12.8	14.3
	3	ND	26.7	23.8	16.3	18.0	14.6
	8	ND	34.8	25.3	13.2	7.5	13.3
GATA-2	1	ND	66.5	36.5	110.6	10.6	26.6
	3	ND	53.0	29.0	68.7	6.9	15.8
	8	ND	57.3	35.4	67.6	7.4	15.9
PU.1	1	ND	8.3	7.4	14.6	11.4	11.5
	3	ND	8.9	6.4	10.1	13.3	12.9
	8	ND	9.8	6.8	10.2	10.9	11.3
Caspase-3	1	ND	85.6	58.6	41.8	9.7	15.4
	3	ND	102.0	56.5	62.0	9.2	11.4
	8	ND	158.1	50.7	63.2	10.0	9.6
Bcl-2	1	ND	99.5	121.7	69.9	17.4	31.9
	3	ND	99.0	118.8	53.5	15.7	27.2
	8	ND	114.6	123.4	44.9	17.8	25.8
Bax	1	ND	222.7	122.5	167.8	55.3	85.7
	3	ND	225.1	99.0	181.7	32.2	69.2
	8	ND	278.4	122.7	156.8	39.7	86.0
Bcl-2/Bax	1	ND	0.4	1.0	0.8	0.3	0.4
	3	ND	0.4	1.2	0.7	0.5	0.4
	8	ND	0.4	1.0	0.9	0.4	0.3
Bax/Bcl-2	1	ND	2.3	1.0	1.2	3.2	2.7
	3	ND	2.3	0.8	1.5	2.1	2.5
	8	ND	2.4	1.0	1.1	2.2	3.3

Abbreviations: Bax, Bcl-2-like protein 4; Bcl-2, B-cell lymphoma; CD, Cluster of differentiation; G, Gap; GATA, globin transcription factor; IkBa, nuclear factor of kappa light polypeptide gene enhancer in B-cells inhibitor, alpha; M, Mitosis; MDS, myelodysplastic syndrome; ND, Not done; NFkB, nuclear factor kappa-light-chain-enhancer of activated B cells; S, Synthesis. Parameters under response to treatment are analyzed by flow cytometry, and results given as MFI (median fluorescnece intensity). For CD markers and cell cycle markers, results are given as % positive cells. LPK is > 90% blasts. Patient 1, 2 and 5 also showed temporal decreased circulating blasts in response to treatment.
