# Supplementary figures and images for: p53 Protein Isoform Profiles in AML: Correlation with Distinct Differentiation Stages and Response to Epigenetic Differentiation Therapy
Source: Cells. 2021 Apr 7;10(4):833. doi: 10.3390/cells10040833 (PMC8068061; doi:10.3390/cells10040833)

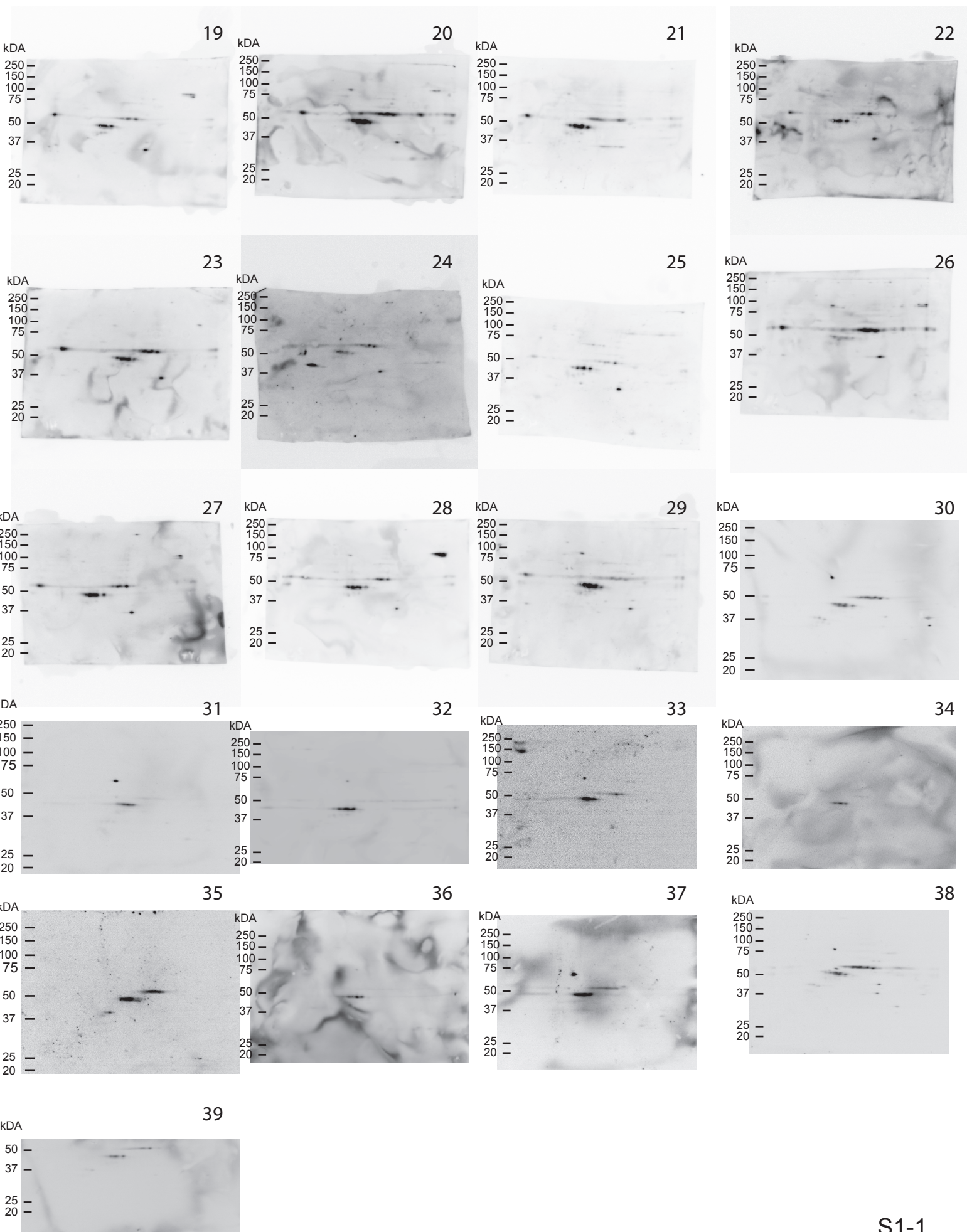

Supplement: Supplementary file 1 [file cells-10-00833-s001.zip › Supplementary data for paper/Supplementary Figure 1-1.pdf]

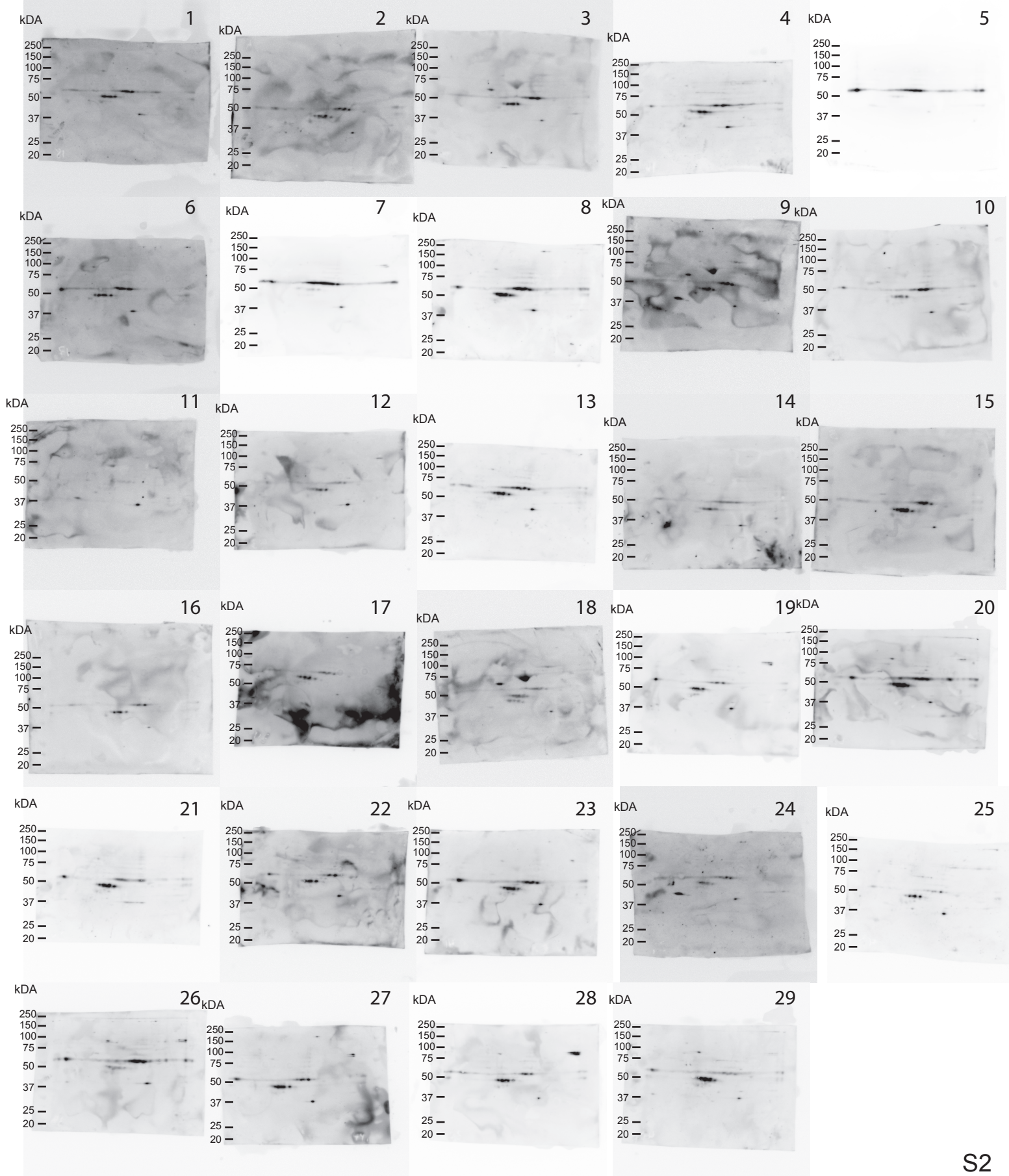

Supplement: Supplementary file 1 [file cells-10-00833-s001.zip › Supplementary data for paper/Supplementary Figure 2.pdf]

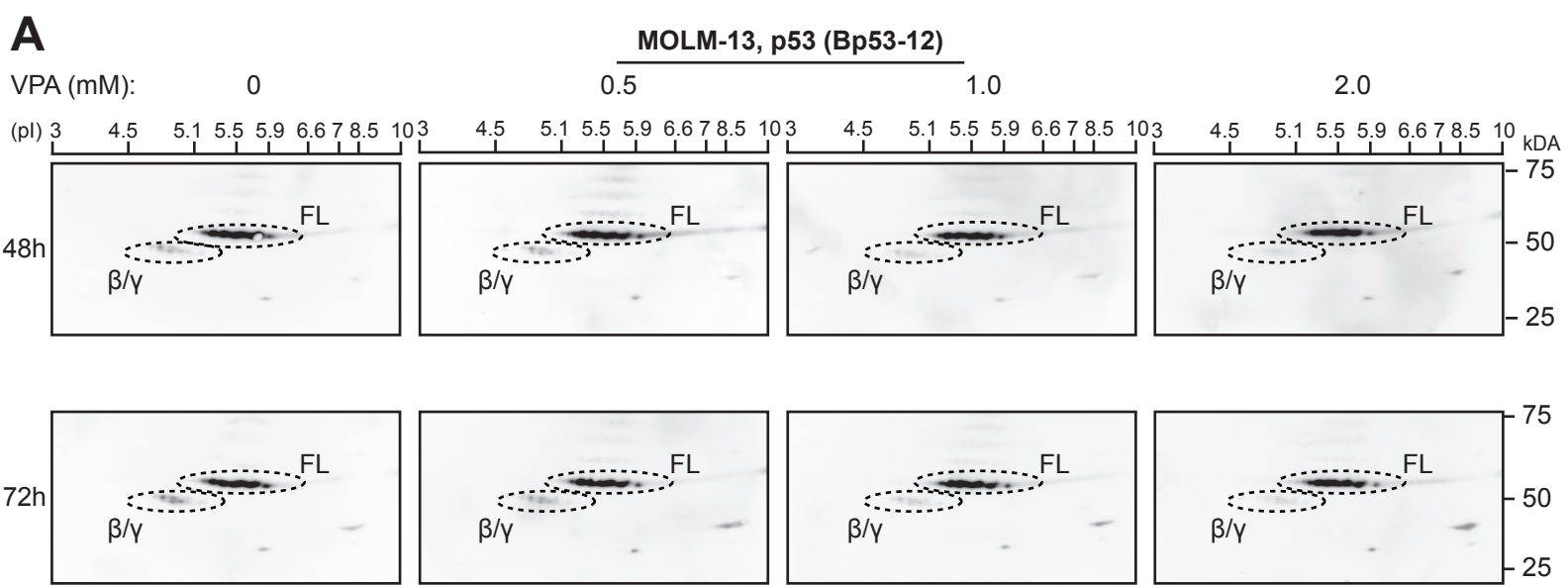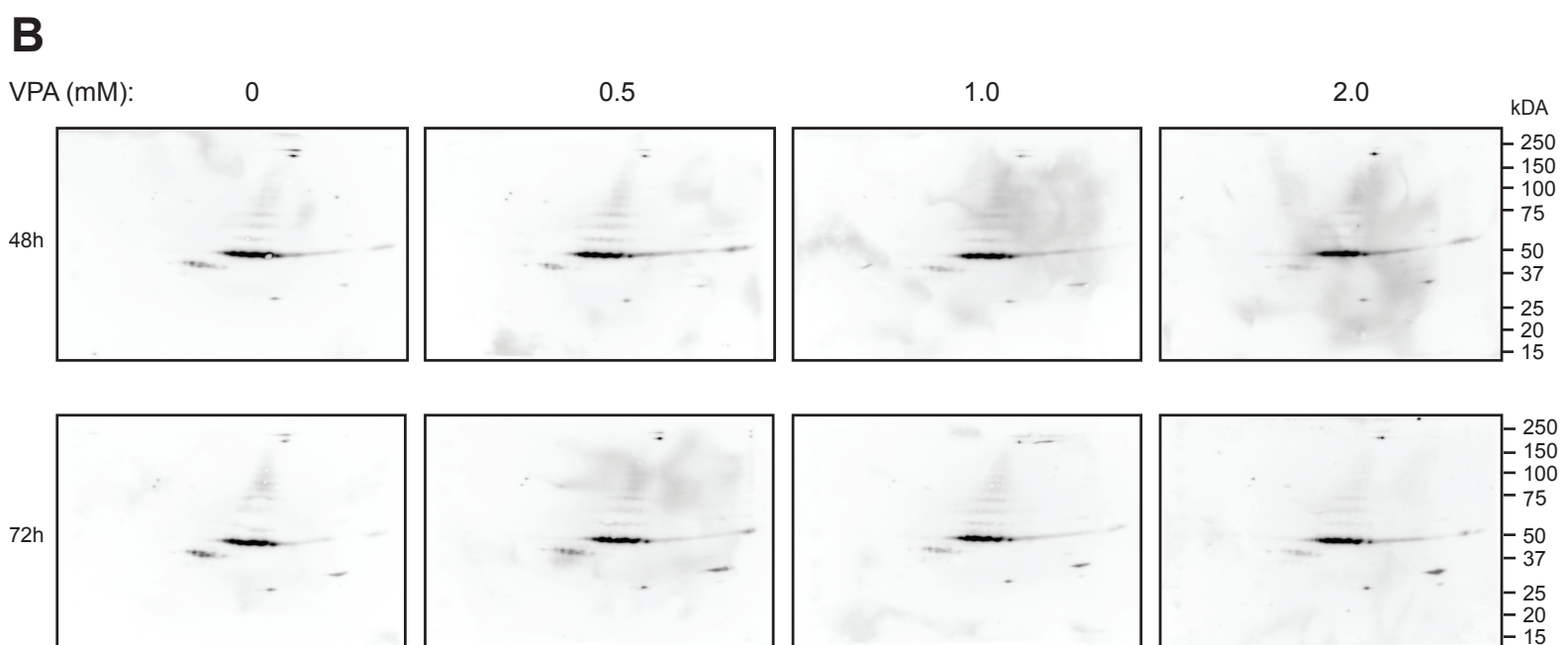

Supplement: Supplementary file 1 [file cells-10-00833-s001.zip › Supplementary data for paper/Supplementary Figure 3-1.pdf]

**A**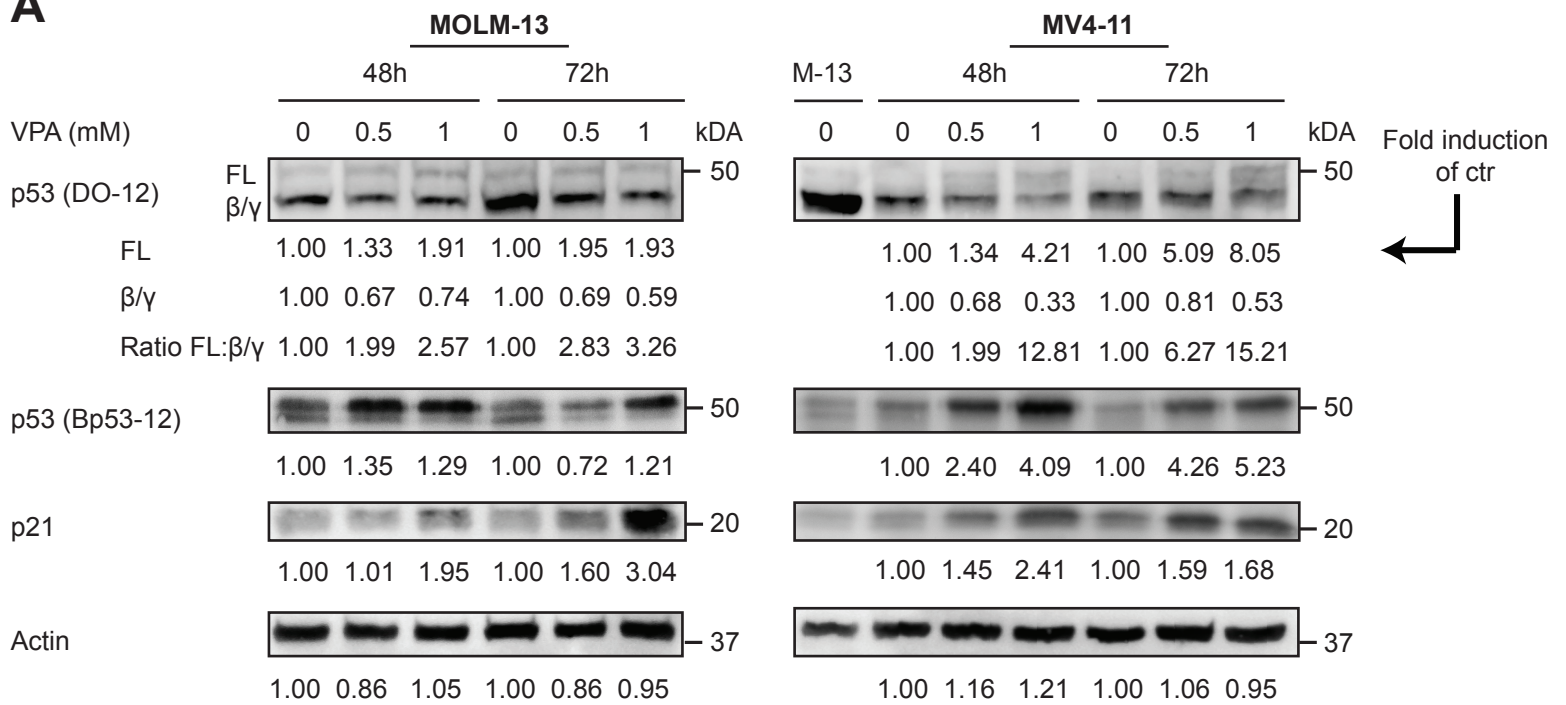**B**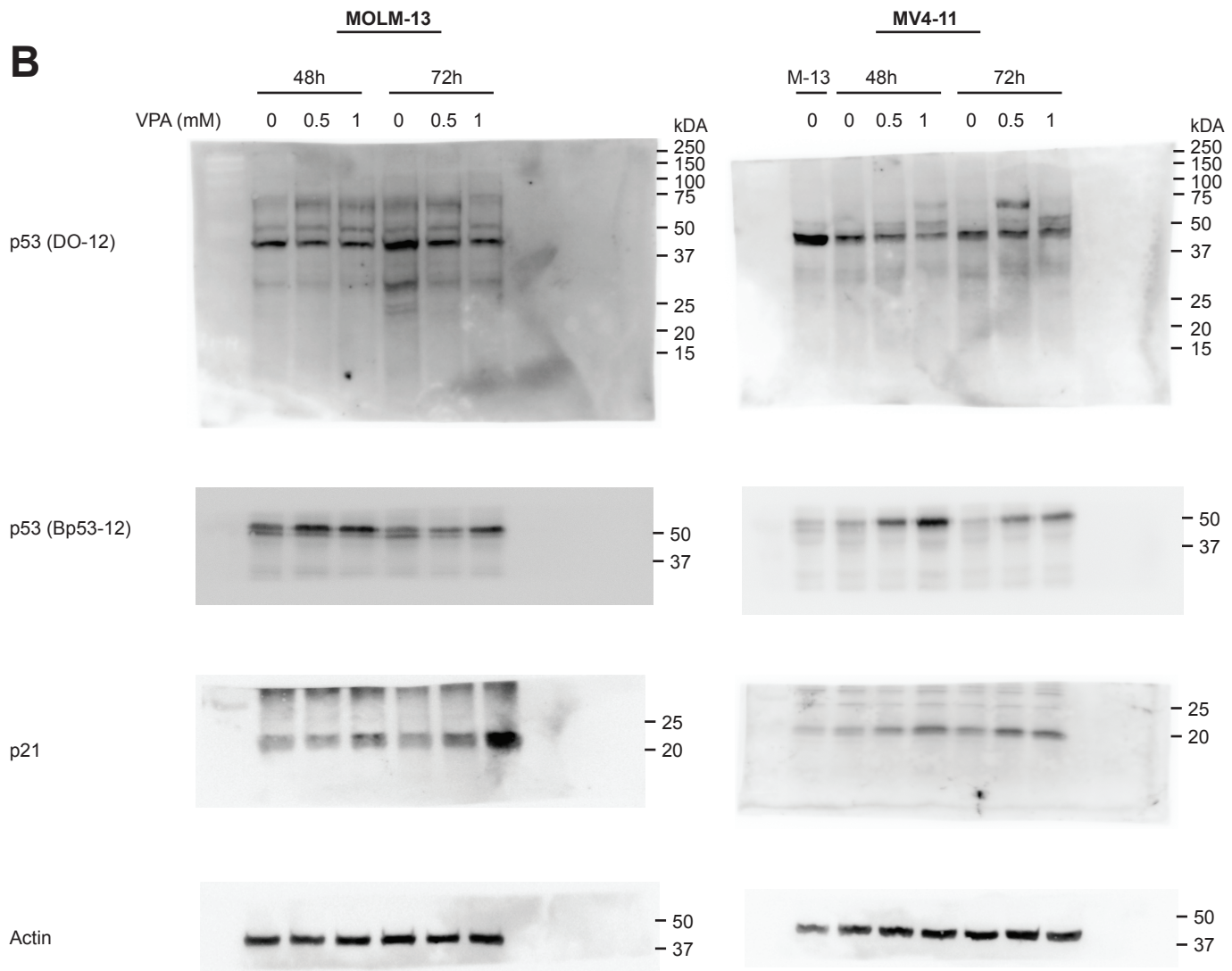

Supplement: Supplementary file 1 [file cells-10-00833-s001.zip › Supplementary data for paper/Supplementary Figure 3-2.pdf]

**A****Samples from AML patients treated with VPA, ATRA and theophylline (*n* = 6)**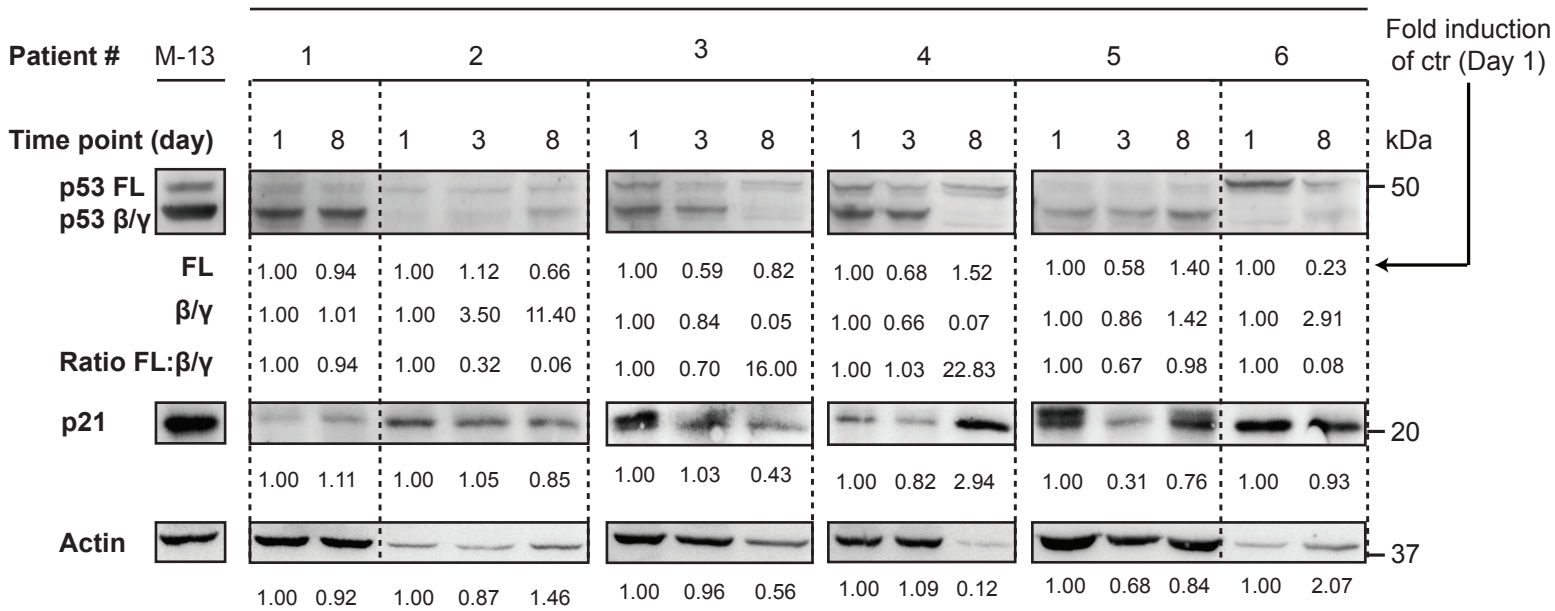**B**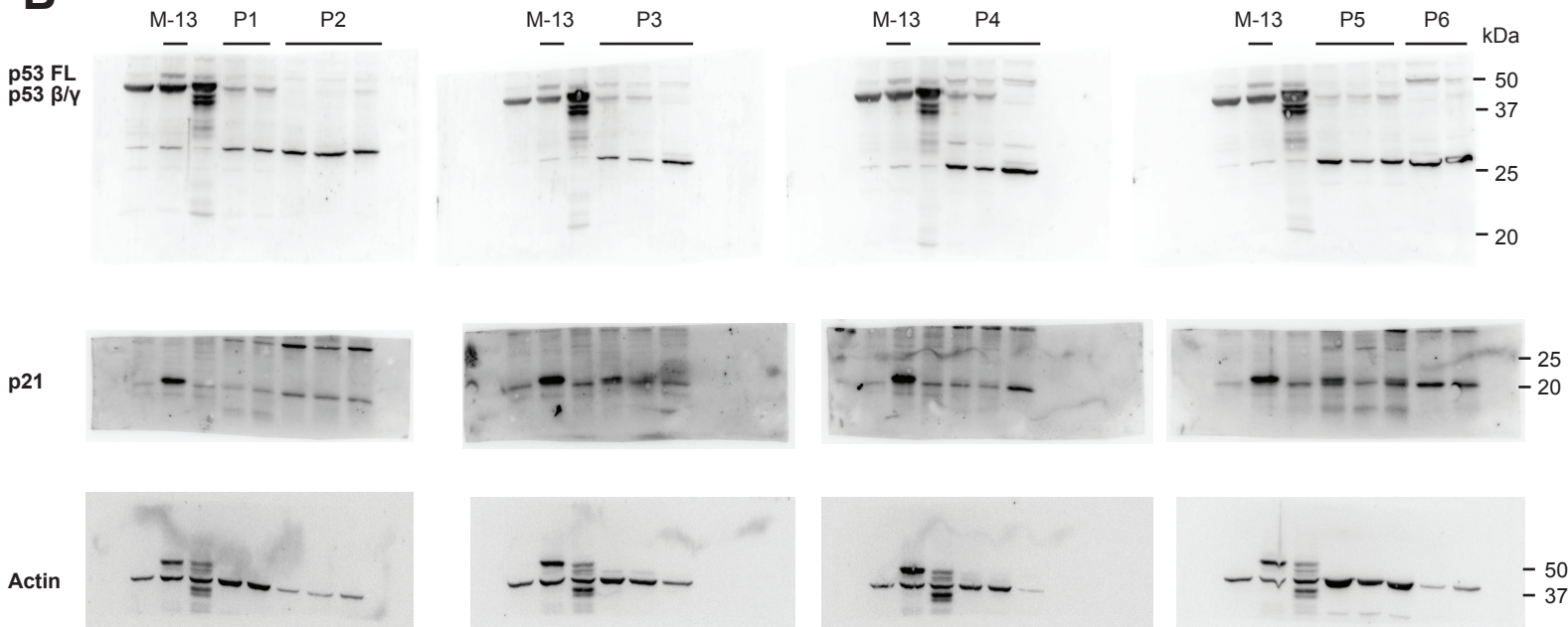

Supplement: Supplementary file 1 [file cells-10-00833-s001.zip › Supplementary data for paper/Supplementary Figure 4.pdf]
